# Transient increase in neuronal chloride concentration by neuroactive aminoacids released from glioma cells

**DOI:** 10.3389/fnmol.2012.00100

**Published:** 2012-11-26

**Authors:** Cristina Bertollini, Emanuele Murana, Luciana Mosca, Maria D'Erme, Federico Scala, Antonio Francioso, Myriam Catalano, Cristina Limatola, Piotr Bregestovski, Silvia Di Angelantonio, Davide Ragozzino

**Affiliations:** ^1^Department of Physiology and Pharmacology, Istituto Pasteur-Fondazione Cenci Bolognetti, Sapienza University of RomeRome, Italy; ^2^Department of Biochemical Sciences, Sapienza University of RomeRome, Italy; ^3^IRCCS NeuromedPozzilli, Italy; ^4^INSERM URM 1106, Aix-Marseille Université Brain Dynamics InstituteMarseille, France; ^5^Center for Life Nano Science at Sapienza, Istituto Italiano di Tecnologia—Sapienza University of RomeRome, Italy

**Keywords:** Cl-Sensor, glioma cells, hippocampus, glutamate, anionic channels

## Abstract

Neuronal chloride concentration ([Cl^−^]_i_) is known to be dynamically modulated and alterations in Cl^−^ homeostasis may occur in the brain at physiological and pathological conditions, being also likely involved in glioma-related seizures. However, the mechanism leading to changes in neuronal [Cl^−^]_i_ during glioma invasion are still unclear. To characterize the potential effect of glioma released soluble factors on neuronal [Cl^−^]_i_, we used genetically encoded CFP/YFP-based ratiometric Cl-(apical) Sensor transiently expressed in cultured hippocampal neurons. Exposition of neurons to glioma conditioned medium (GCM) caused rapid and transient elevation of [Cl^−^]_i_, resulting in the increase of fluorescence ratio, which was strongly reduced by blockers of ionotropic glutamate receptors APV and NBQX. Furthermore, in HEK cells expressing GluR1-AMPA receptors, GCM activated ionic currents with efficacy similar to those caused by glutamate, supporting the notion that GCM contains glutamate or glutamatergic agonists, which cause neuronal depolarization, activation of NMDA and AMPA/KA receptors leading to elevation of [Cl^−^]_i_. Chromatographic analysis of the GCM showed that it contained several aminoacids, including glutamate, whose release from glioma cells did not occur via the most common glial mechanisms of transport, or in response to hypoosmotic stress. GCM also contained glycine, whose action contrasted the glutamate effect. Indeed, strychnine application significantly increased GCM-induced depolarization and [Cl^−^]_i_ rise. GCM-evoked [Cl^−^]_i_ elevation was not inhibited by antagonists of Cl^−^ transporters and significantly reduced in the presence of anion channels blocker NPPB, suggesting that Cl^−^ selective channels are a major route for GCM-induced Cl^−^ influx. Altogether, these data show that glioma released aminoacids may dynamically alter Cl^−^ equilibrium in surrounding neurons, deeply interfering with their inhibitory balance, likely leading to physiological and pathological consequences.

## Introduction

In the central nervous system (CNS), a tight regulation of intracellular chloride concentration ([Cl^−^]_i_) is important for a number of cellular functions, including the stabilization of resting membrane potential, the regulation of both intracellular pH and cell-volume (Pasantes-Morales et al., 2006; Suzuki et al., 2006) and the strength and polarity of γ-aminobutyric acid (GABA) and glycine-mediated neurotransmission (Payne et al., 2003).

Several different pathways allow Cl^−^ movements across neuronal membranes, determining Cl^−^ equilibrium. Among these are the ligand-gated anion channels (GABA_A_ and glycine receptors), the cation-chloride cotransporters (KCC2 and NKCC1) and a variety of Cl^−^ channels, including Ca^2+^-, volume-, and voltage-activated Cl^−^ channels (Payne et al., 2003; Suzuki et al., 2006; Jentsch, 2008; Deisz et al., 2011).

Neuronal Cl^−^ equilibrium is subject to alterations both in physiological and pathological conditions (Staley et al., 1995; Planells-Cases and Jentsch, 2009; Doyon et al., 2011) and abnormal Cl^−^ homeostasis is associated with neuronal trauma or brain disorders (De Koninck, 2007). Particularly, in epilepsy, the alteration of Cl^−^ homeostasis is a widespread phenomenon, contributing to neuronal hyperexcitability (Palma et al., 2006; Barmashenko et al., 2011; Conti et al., 2011). The reported changes in neuronal Cl^−^ equilibrium affect the inhibitory power of GABA/glycine, causing its switching to excitatory action. In addition, neuronal transmembrane Cl^−^ gradient may be altered by glutamatergic stimulation, through the activation of anionic channels or changes in Cl^−^ transporters activity (Van Damme et al., 2003; Slemmer et al., 2004; Kitamura et al., 2008). In the brain, a similar condition may occur in case of excessive glutamate release, under intense synaptic activity (Fiumelli et al., 2005; Fiumelli and Woodin, 2007) or reduced glutamate uptake by astrocytes (Danbolt, 2001). Indeed, even modest increases in extracellular glutamate concentration can alter synaptic transmission (Araque et al., 1999) and reduced activity of glial glutamate transporters has been suggested to contribute to and exacerbate a number of neurological conditions, including stroke, epilepsy, cerebral ischaemia, amyotrophic lateral sclerosis, and others (O'Shea, 2002).

A dysfunction in glutamate transport has also been reported in malignant gliomas (Ye et al., 1999). The comparison of glutamate transport into astrocytes vs. their malignant counterparts showed that gliomas behave opposite to astrocytes, releasing glutamate rather than sequestering it (Ye and Sontheimer, 1999). Moreover, when glioma cells were co-cultured with neurons, the released glutamate activated neuronal NMDA receptors, resulting in excitotoxic cell death, suggesting that the release of excitotoxic concentrations of glutamate may promote tumor expansion (Sontheimer, 2003). In addition, glutamate, as well as other aminoacids (GABA, glycine, serine) may be released, in the brain, by various cell types during volume changes (Pasantes-Morales et al., 2006), occurring upon cell migration, edema, or during glioma cell invasion (Ordaz et al., 2004; Pasantes-Morales and Vázquez-Juárez, 2012).

In this study we analyzed how glutamate or other aminoacids, released by glioma cells cause an alteration of Cl^−^ homeostasis. Taking advantage of a genetically encoded CFP/YFP-based ratiometric chloride sensor (Cl-Sensor) (Markova et al., 2008; Waseem et al., 2010), we investigated how neuronal [Cl^−^]_i_ is dynamically regulated by diffusible factors released by cultured glioma cells.

We report that glutamate released from glioma cells triggers neuronal Cl^−^ rise through the activation of ionotropic glutamate receptors, cell depolarization and activation of anionic channels. Such mechanism may partially explain how glioma invasion causes local dynamic Cl^−^ changes and neuronal Cl^−^ equilibrium dysfunction.

## Materials and methods

### Animals

Animal procedures were conducted in accordance with the international guidelines on the ethical use of animals from the European Communities Council Directive of 24 November 1986 (86/609/EEC). C57BL/6 (Charles River Laboratory) of either sex was used.

### Cell cultures

The glioblastoma patient derived cell line MZC (M.Z.C., kindly provided by Dr. Antonietta Arcella, Neuromed, Italy, Sciaccaluga et al., 2010) was grown in Dulbecco's Modified Eagle Medium (DMEM, GIBCO) supplemented with 10% heat-inactivated Fetal Bovine Serum (FBS), 100 IU/ml penicillin G and 100 μg/ml streptomycin at 37°C in a 5% CO_2_ humidified atmosphere. Medium was changed twice a week and the cells were sub-cultivated when confluent and used between 20th and 40th passage when they had reached 80% confluence.

Primary hippocampal neuronal cultures were obtained from postnatal day 0–2 (P0-P2) C57BL/6 mice. Briefly, after careful dissection from diencephalic structures, the meninges were removed under a dissection microscope and the hippocampi were collected in ice-cold Hank's balanced salt solution (HBSS, GIBCO Invitrogen) and chopped and digested in 1.25 mg/ml trypsin for 15 min at 37°C. Subsequently, cells were mechanically dissociated and plated at a density of 10^5^ in poly-L-lysine coated round glass coverslips (12 mm diameter) put into 24-well culture plates in serum-free Neurobasal medium, supplemented with 2% B27, 0.5 mM L-Glutamine and 100 μg/ml gentamicin (culture medium). Finally, cells were kept at 37°C in 5% CO_2_ for 10–12 days with a twice a week medium replacement (1:1 ratio). With this method we obtained 60–70% neurons, 30–35% astrocytes, and 4–5% microglia, as determined with β-tubulin III, glial fibrillary acidic protein, and isolectin IB4 staining (Lauro et al., 2010).

Primary cortical glial cells were prepared from P0–P2 -old mice. Cerebral cortices were chopped and digested in 30 U/ml papain for 40 min at 37°C followed by gentle trituration. The dissociated cells were washed, suspended in DMEM with 10% FBS (Invitrogen) and 2 mm l-glutamine and plated at a density of 9–10 × 105 in 175 cm^2^ cell culture flasks. At confluence (10–14 DIV), glial cells were shaken for 2 h at 37°C to remove microglial cells. These procedures gave almost pure astrocytes cell population (4–6% of microglia contamination), as verified by staining with glial fibrillary acidic protein and isolectin IB4 (Rosito et al., 2012). Astrocytes were re-plated and used for experiments.

The human retroviral packaging cell line HEK 293 stably expressing the rat flip variant of wild-type glutamate receptor 1 (GluR1-HEK cells) was grown in DMEM with geneticin (0.5 mg/ml), Glutamax-I/10% FBS 1% penicillin/streptomycin, 5% CO2 (37°C). Cells were plated onto 12 mm round coverslips at a density of 104 cells/cm^2^.

### Glioma conditioned medium (GCM)

Confluent MZC cultures were incubated for 4 h with 10 ml of filtered normal external solution (NES) containing (in mM): 140 NaCl, 2.8 KCl, 2 CaCl_2_, 2 MgCl_2_, 10 HEPES-NaOH, and 10 glucose (pH 7.32; 300 ± 5 mOsm). This GCM was then centrifuged, pH adjusted to 7.32 and used for the experiments. The hyperosmotic glioma conditioned medium (HyperGCM) was obtained by incubating glioma cells with the following external solutions in (mM): NaCl 159.6, KCl 3.2, MgCl_2_ 2.28, CaCl_2_ 2.28, HEPES-NaOH 11.4, glucose 11.4 (114% concentrated NES); or NaCl 154, KCl 2.8, MgCl_2_ 2, CaCl_2_ 2, HEPES-NaOH 10, glucose 25. Both solutions, adjusted at pH 7.32, were isoosmotic compared to DMEM (340 ± 10 mOsm).

### Hippocampal cultures transfection

Twenty Four hours before transfection, 50% of the neuronal growth medium was replaced with fresh culture medium. For transfection 100 μl of MEM was mixed with 2 μl of Magnetofection NeuroMag (OZ Bioscience, France) and 1 μg of the cDNA of Cl-Sensor (Markova et al., 2008). The mixture was incubated for 15–20 min at room temperature and thereafter distributed dropwise over the neuronal culture. Cells were then placed on a magnetic plate (OZ Bioscience) and incubated for 15 min at 37°C. Transfection was terminated by substitution of 50% of incubation solution with the fresh culture medium. Cells were used for the experiments 24–76 h after transfection.

### Fluorescence determinations

Fluorescence images were acquired at room temperature (22–24°C) using a customized digital imaging microscope. Excitation of cells at various wavelengths was achieved using a 1-nm-bandwidth polychromatic light selector (Till Polychrome V) equipped with a 150 W xenon lamp (Till Photonics, Germany). For the use of Cl-Sensor the optic system is equipped with an excitation filter D480/30x, an emission filter D535/40m and a dichroic mirror 500DRLP (500 nm) (Omega Optics, USA); for the use of FURA-2AM we used a conventional set of filters (SP410, 510/40 m; 400DCLP 400 nm; Omega Optics, USA). Fluorescence was visualized using an upright microscope (Axioskope) equipped with a 40 × water-immersion objective (Achroplan CarlZeiss, USA) and a digital 12 bit CCD camera system (SensiCam, PCO AG, Germany); images were acquired on a computer via a double-coaxial cable. All peripheral hardware control, image acquisition and image processing were achieved using customized software TillVision v. 4.0 (Till Photonics, Germany).

Cells expressing Cl-Sensor were excited alternatively at 445 and 485 nm wavelengths (50 ms, 0.1 Hz). [Cl^−^]_i_ changes are expressed as a ratio of background subtracted *F*_445_ over *F*_485_ (*R* = *F*_445_/*F*_485_). Before starting all the experiments, we evaluated the basal Cl^−^ level in each neuron, through the emission spectrum (for methods see Markova et al., 2008; Bregestovski et al., 2009). Cells whose shape of emission spectrum corresponded to high [Cl^−^]_i_, were not considered.

The Ca^2+^ sensitive indicator Fura-2 was used to monitor changes in the intracellular calcium level in hippocampal neurons. Cells (10–12 DIV) were loaded by adding cell permeant acetoxymethyl (AM) derivatives dye (4 μM) and incubating dishes at 37°C for 45 min. Fura-2AM loaded cells were excited alternatively with dual wavelengths of 340 and 380 nm (20 ms; 0.2 Hz). Intracellular Ca^2+^ changes are expressed as a ratio between fluorescence emitted after excitation at 340 nm (*F*_340_) and after excitation at 380 nm (*F*_380_) [Fluorescence Ratio (*R*) = *F*_340_/*F*_380_].

### Patch-clamp recordings

Whole-cell patch-clamp recordings were performed using a Axopatch 200A amplifier, Digidata 1440 digitizer, on a PC running pCLAMP software (all from Molecular Devices, Foster city, CA). Data were acquired at 10 kHz and filtered at 2 kHz. Patch electrodes were prepared from borosilicate glass using a vertical electrode puller (PC10, Narishige, Japan) to produce tip openings of 1–2 μm (4–6 MΩ). Electrodes were filled with an intracellular solution containing (in mM): 135 CsMetSO_4_, 2 MgCl_2_, 2 MgATP, 0.3 NaGTP, 10 HEPES, 0.5 EGTA; pH 7.3 with CsOH. Recording were performed in normal extracellular solution containing (in mM): 140 NaCl, 2.5 KCl, 2 CaCl_2_, 2 MgCl_2_, 10 HEPES–NaOH, and 10 glucose (pH 7.3 adjusted with NaOH, osmolarity 300 mOsm). In voltage-clamp mode of recording cells were held at −70 mV; in current-clamp experiments, the voltage was recorded while cells were injected with holding current (30–230) pA. Glutamate concentration–current curves were constructed by applying to each HEK-AMPA cell (held at −50 mV) four or five different concentrations of agonist (0.01–1 mM) at 60–120 s intervals, in the presence of 25 μM cyclothiazide, to prevent GluR1 receptor desensitization (Fucile et al., 2006), and normalizing the current response to the plateau value, tested in all cells (corresponding to 1 mM). For each cell, the concentration-current curve was best-fitted using Origin 7 (OriginLab Corp., Northampton, MA, USA) to the Hill equation: *I*_norm_ = 1/(1 + *EC*^*n*H^_50_/[Glutamate]^*n*H^) where *I*_norm_ is the normalized current response, EC_50_ is the agonist concentration yielding half-maximal current response and *n*_H_ is the Hill coefficient. These curves were averaged and used to calculate the mean EC_50_ for glutamate.

### Experimental procedures and drugs application

During fluorescence or electrophysiological measurements, cells were continuously superfused with external solution, using a gravity driven perfusion system, positioned 50–100 μm from the cell. Unless otherwise indicated, all experiments were conducted in normal extracellular solution. Concentration response curves in HEK-AMPA cells were performed with a computer controlled fast perfusion system (Warner Instruments, USA). Tetrodotoxin (TTX, 1 μM) was routinely dissolved in the extracellular medium. Low Cl^−^ medium contained: 147.5 mM Na-Gluconate, 2.8 mM K-Gluconate, 2 mM MgCl_2_, 2 mM CaCl_2_, 10 mM Glucose, and 10 mM HEPES-NaOH (pH 7.32; 300 ± 5 mOsm). The fluorescence signal was monitored online and stable baseline responses were recorded for at least 4 min before applying drugs. Tetrodotoxin citrate (TTX), D-2-amino-5-phosphonopentanoic (D-APV), 2,3-dihydroxy-6-nitro-7-sulfamoyl-benzo[f]quinoxaline-2,3-dione (NBQX), glutamate, GABA, strychnine hydrochloride, and bicuculline methochloride were purchased from Abcam (UK). 5-Nitro-2-(3-phenylpropylamino)benzoic acid (NPPB) and DL-*threo*-β-Benzyloxyaspartic acid (TBOA) were purchased from Tocris (UK). All other drugs used were purchased from Sigma-Aldrich, Milan, Italy.

NPPB, Sulfasalazine (SAS), and TBOA were dissolved in Dimethyl sulfoxide (DMSO) and used at 1/1000 dilution and the same concentration of solvent was used in control condition. Unless otherwise indicated, AP5, NBQX, and strychnine were applied to the cells only in co-application with GCM. The duration of NPPB pretreatment (3–4 min) was set to allow stabilization of fluorescence ratio after the change induced by drug application.

### Chromatographic analysis

Aminoacids were determined in supernatants by reversed-phase High Performance Liquid Chromatography (HPLC) following *o*-phthaldialdehyde (OPA) derivatization in the presence of 2-mercaptoethanol. 100 μL of samples or standards were mixed 1:1 (v/v) with freshly prepared OPA/2-mercaptoethanol derivatizing solution (3,5 mg of OPA in 50 μL of 95% ethanol, diluted with 5 mL of borate buffer, pH 10.4, plus 10 μL of 2-mercaptoethanol, protected from light) and injected within 1 min onto the column (Hirschberger et al., 1985). The HPLC consisted of a Waters apparatus equipped with a 600 pump and pump controller, a Rheodyne injection valve with a 50 μL loop, a Nova-Pack C18 column (reverse phase, 3.9 × 150 mm, 4 μm particle size, thermostated at 41°C with a 10 mm guard column of the same material matrix) and a Shimadzu RF-551 fluorometric detector, operating at λ_ex_ 360 nm and λ_em_ 455 nm. The elution was performed at a flow rate of 1 mL/min, with solvent A being 25 mM phosphate buffer, pH 7.0, containing 3% THF, and solvent B 25 mM phosphate buffer, pH 7.0, containing 40% CH_3_CN and 3% THF. The mobile phase was run isocratically at 5% B for the first 5 min, then solvent B linearly increased to 27% in 10 min, remained for 20 min at 27% B and finally increased linearly to 100% in 1 min. At 40 min, buffer B was returned to 5% and the column was allowed to equilibrate for 10 min. Total run time was 50 min per sample. Peak identification was performed on the basis of the retention time and by spiking the sample with appropriate standards, whereas aminoacid quantitation was performed by automatic peak area integration using dedicated software (Millennium^32^, Waters). Results are expressed as μmoles of aminoacid per L of sample.

### Data analysis and statistics

Data are analyzed with Clampfit 10 (Molecular Devices) Statistical significance (*p* < 0.05) was determined using the paired or unpaired Student's *t*-test (Origin software; Microcal Software, Northampton, MA). Data are reported as means±standard error of the mean (SEM) and “*n*” values refers to the number of neurons examined. All studies involved different culture sets. Each set of experiments presented was internally controlled, using similar numbers of control coverslips to compare with each experimental condition.

## Results

### GCM evokes rapid and reversible increase in [Cl^−^]_i_ via a glutamatergic mechanism in cultured hippocampal neurons

**Figure 1:**
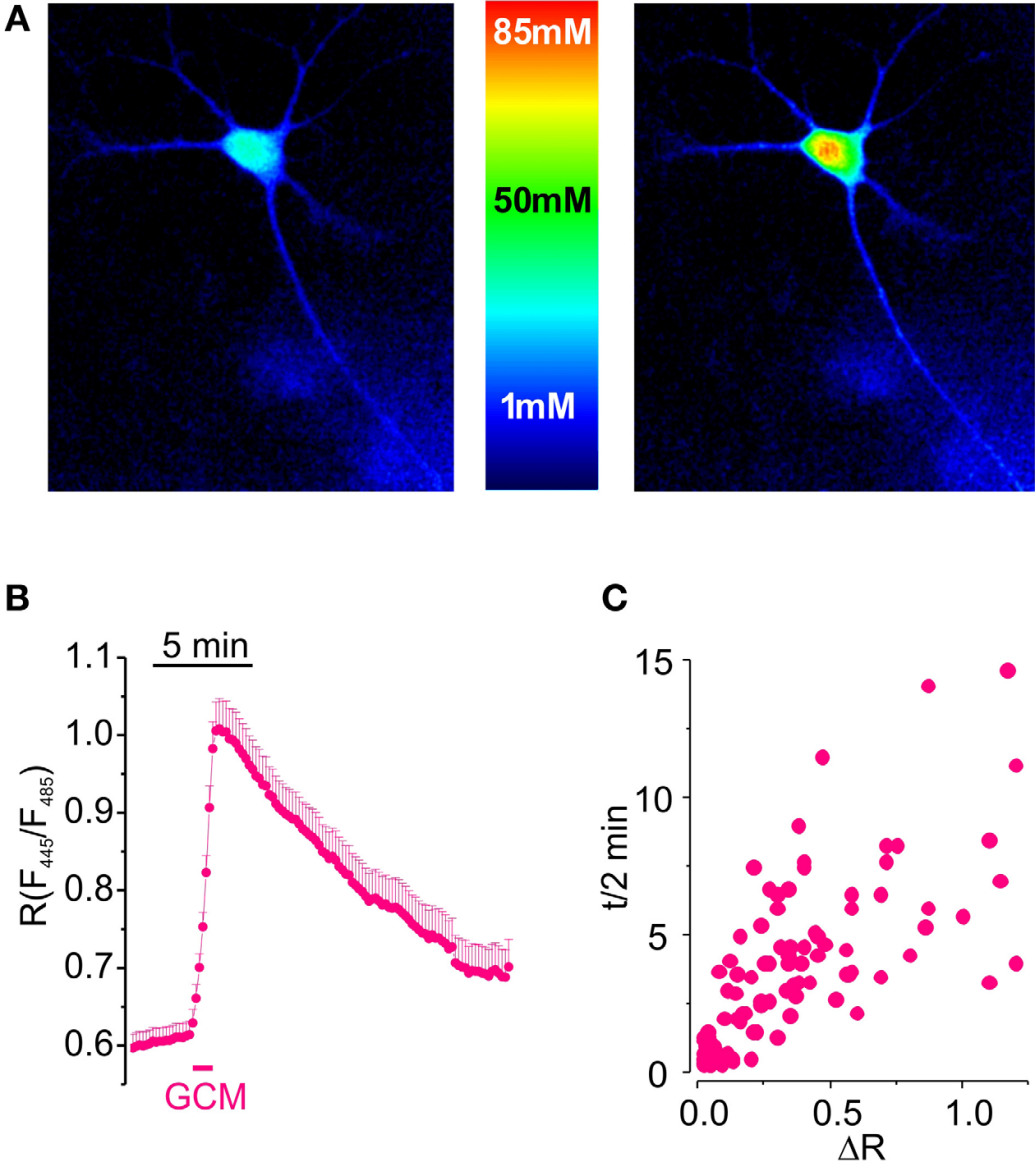
**Glioma conditioned medium evokes a transient increase in [Cl^−^]_i_ in hippocampal cultured neurons. (A)** Visualization of [Cl^−^]_i_ increase induced by GCM in hippocampal neurons expressing Cl-Sensor. **(B)** Time course of fluorescence ratio increase induced by the exposure of hippocampal neurons transfected with Cl-Sensor to GCM (1 min; *n* = 108). **(C)** Relationship between the amplitude and the duration of [Cl^−^]_i_ transients in hippocampal neurons; each point represents a single determination of peak amplitude (ΔR) and time of half decay (*t*/2) of GCM effect.

In order to assess whether substances released from glioma cells may alter neuronal chloride homeostasis, we used a CFP/YFP-based ratiometric Cl-Sensor expressed in primary hippocampal neuronal cultures (Figure 1A). A glioma conditioned medium (GCM), prepared exposing cultured MZC glioma cells to NES, was applied to neurons transfected with Cl-Sensor and changes in fluorescence ratio (*R*), associated to [Cl^−^]_i_ variations, were monitored over time.

Results indicate that GCM applied for 1 min onto neurons expressing Cl-Sensor evokes a rapid and reproducible increase in fluorescence ratio (*R*) from 0.63 ± 0.02 to 0.94 ± 0.04 (Δ*R* = 0.32 ± 0.03, *n* = 153, *p* < 0.01), indicating a strong rise in [Cl^−^]_i_ (Figure 1B). *R* increase was followed by a slow recovery of basal fluorescence, with an average half decay time (*t*/2) of 3.5 ± 0.3 min (*n* = 123). As shown in Figure 1C, the duration of *t*/2 increased with peak amplitude of response to GCM.

**Figure 2:**
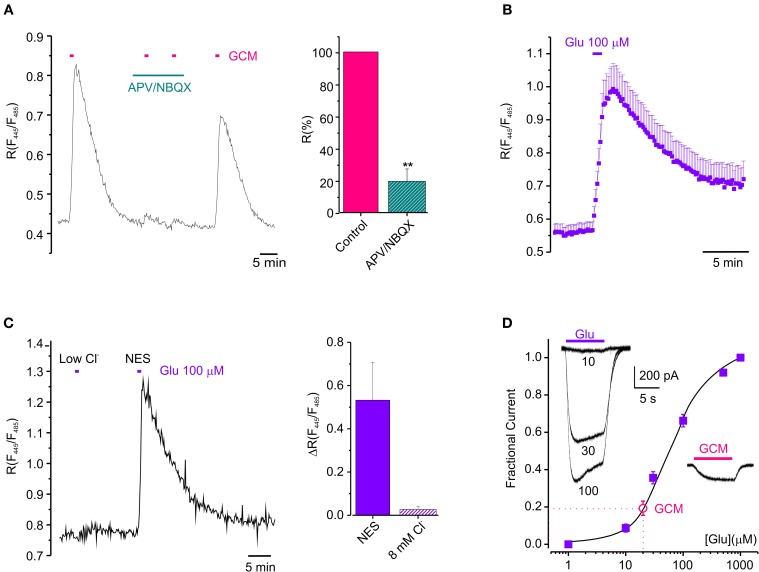
**Glutamatergic mechanism GCM-evoked [Cl^−^]_i_ increase. (A)** Effect of ionotropic glutamate receptors antagonists on GCM-evoked [Cl^−^]_i_ increase. Left, fluorescence trace from an example neuron showing the effect of application of GCM alone and in the presence of APV (20 μM) and NBQX (10 μM). Right, bar chart represents the average of fluorescence response elicited by GCM in the presence of APV (20 μM) and NBQX (10 μM) expressed as the % of control (*n* = 5 neurons). **(B)** Time course of the effect of 100 μM glutamate on the fluorescence ratio in Cl-Sensor transfected hippocampal neurons (*n* = 28). **(C)** Fluorescence response to glutamate in control and in gluconate-based low Cl^−^ medium (8 mM); Left, fluorescence trace from an example neuron showing the effect of application of glutamate (100 μM) in control and in gluconate-based low Cl^−^ medium (8 mM). Right, bar chart represents the average of fluorescence response elicited by glutamate in the absence of external Cl^−^ expressed as the % of control (*n* = 6 neurons). **(D)** Concentration-current response curve for glutamate obtained in HEK-AMPA cells (filled squares, ■; *n* = 12). Empty circle (°) represents the average amplitude of the current evoked by GCM application in the same cells, indicative of a glutamate concentration in GCM of 20 ± 2 μM. (*n* = 6). Inset, typical whole-cell currents evoked by glutamate (10-30-100 μM, left) and GCM (right) on HEK-AMPA cells **(A**–**D)**, horizontal bars represents drugs application as indicated. Data are shown as mean values ± SEM. ^**^p < 0.01.

Since glioma cells may release glutamate in the extracellular space (Ye and Sontheimer, 1999; Buckingham et al., 2011) we wondered whether GCM-induced neuronal [Cl^−^]_i_ rise could be triggered by the activation of ionotropic glutamate receptors on neurons (see Slemmer et al., 2004). To address this issue, we treated hippocampal neurons with NMDA and AMPA receptors antagonists (APV 20 μM; NBQX 10 μM; 3 min), before exposing them to GCM. As shown in Figure 2A, APV and NBQX strongly reduced GCM-induced responses, giving an average decrease of Δ*R* by 81 ± 8% of control (*n* = 5, *p* < 0.01), indicating that GCM leads to a rapid increase of [Cl^−^]_i_, by a glutamatergic mechanism. Consistently, the exposure of Cl-Sensor expressing neurons to glutamate (100 μM) for 1 min evoked a rapid and reversible increase in *R* from 0.56 ± 0.02 to 1.01 ± 0.09 (Δ*R* = 0.45 ± 0.08, *n* = 28, *p* < 0.01), similar to that induced by the application of GCM (Figure 2B). It has been reported that glutamate can cause changes both in [Cl^−^]_i_ and [pH]_i_ in neurons (Wang et al., 1994; Metzger et al., 2002) and Cl-Sensor is known to be sensitive to pH (Markova et al., 2008). Thus, we monitored fluorescence response to glutamate in control and in gluconate-based low Cl^−^ medium (8 mM, similar to the one measured in Cl-Sensor expressing neurons), to exclude that the fluorescence transients could depend on the effect of intracellular acidification on Cl-Sensor properties. The removal of external Cl^−^ abolished *R* increase in response to glutamate (Δ*R* = 0.03 ± 0.01, *n* = 6, *p* < 0.05), suggesting that, in our experimental conditions, the glutamate-induced fluorescence ratio increase truly represents [Cl^−^]_i_ rise (Figure 2C).

Then, in order to functionally evaluate the concentration of glutamate present in GCM, we constructed a concentration-current response curve for glutamate in HEK cell lines stably expressing the GluR1 subunit of AMPA receptors (HEK-AMPA cells) (Fucile et al., 2000), using five different glutamate concentrations and applying GCM on the same cell. Best fits of concentration-current response curves were averaged and the mean EC_50_ for glutamate was 59.4 ± 0.6 μ M (*n*_H_ = 1.30 ± 0.01, *n* = 24). The amplitude of currents evoked by GCM application in the same cells were fitted in the curve, indicating a glutamate concentration of 20 ± 2 μM (Figure 2D *n* = 6). Consistently, the application of 20 μM glutamate to neurons transfected with Cl-Sensor was sufficient to cause a transient increase in [Cl^−^]_i_ (Δ*R* = 0.06 ± 0.01, *n* = 3; not shown).

Altogether, these data suggest that glutamate released from glioma cells triggers neuronal Cl^−^ rise through the activation of ionotropic glutamate receptors.

### Glioma conditioned medium contains excitatory and inhibitory aminoacids

**Figure 3:**
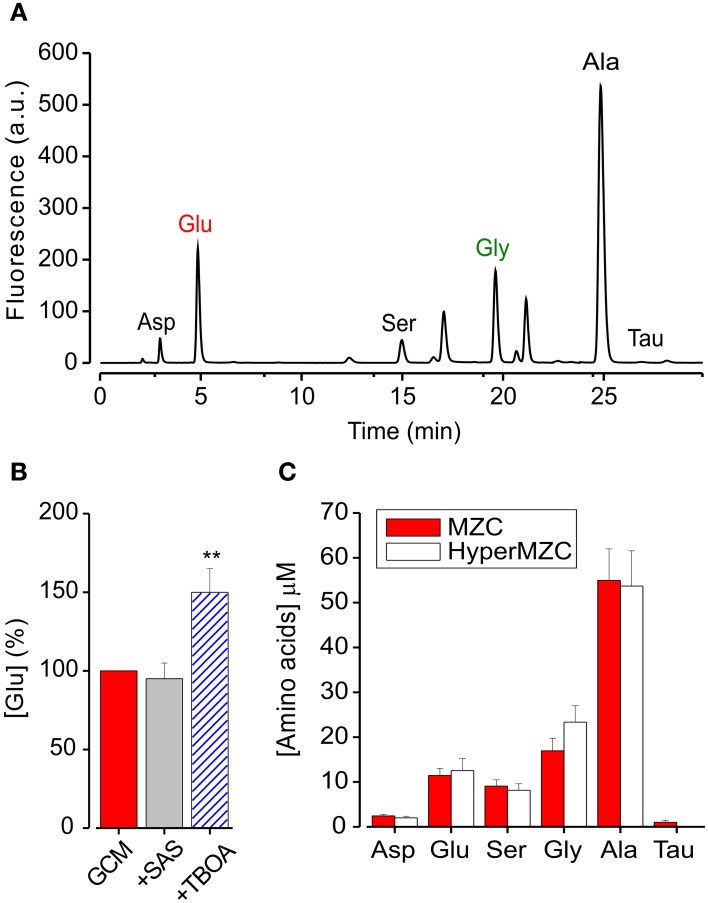
**GCM contains excitatory and inhibitory aminoacids. (A)** Representative chromatogram of aminoacids present in glioma conditioned mediums: Asp, aspartate; Glu, glutamate; Ser, serine; Gly, glycine; Ala, alanine; Tau, taurine; HPLC conditions are described in the text. **(B)** Effects of glutamate transport antagonists on GCM glutamate release. Histogram represents the average of four experiments where GCM was prepared in the presence of SAS (250 mM) or TBOA (100 mM). Glutamate concentration is expressed in % of [glu] in parallel control GCM. **(C)** Effect of osmotic stress on aminoacids release from MZC cells. Histogram represents the concentration of identified aminoacids present in GCM prepared from hyperosmotic (empty columns; *n* = 5) or control NES (filled columns; *n* = 10). ^**^p < 0.01.

We used the High-Performance Liquid Chromatography (HPLC) to quantify the amount of glutamate contained in GCM and identify other aminoacids eventually present. Figure 3A shows a representative chromatogram of the aminoacids contained in GCM. The HPLC analysis confirmed that glioma cells release a significant amount of glutamate, in addition to several other aminoacids in the micromolar range, including alanine, aspartate, serine, and glycine (Behrens et al., 2000). Table 1 summarizes the concentration of the aminoacids present in the GCM. Among these aminoacids, we highlighted both excitatory (glutamate, aspartate, glycine, serine) and inhibitory (glycine, taurine) neuroactive compounds. In particular, glutamate seems to be the most relevant excitatory neurotransmitter, with an average concentration in GCM of 10.6 ± 1.7 μ M (*n* = 11). On the other hand, among inhibitory neurotransmitters, glycine is by far the most representative, as its concentration in GCM is appreciably above the level of taurine or GABA, the latter being undetectable.

**Table 1:** **Concentration of identified aminoacids in glioma and astrocyte-conditioned medium; table summarizes the concentration of aminoacids determined in GCM (*n* = 11) and astrocyte conditioned medium (ACM) (*n* = 4) expressed in μ M**.

**AA**	**Concentration in GCM (μ M)**	**Concentration in ACM (μ M)**
Aspartate	2.3 ± 0.3	6.7 ± 4.1
Glutamate	10.6 ± 1.7	1.5 ± 0.9
Serine	8.5 ± 1.4	31.6 ± 10.1
Glycine	16.0 ± 2.7	22.3 ± 6.0
Alanine	51.7 ± 7.2	32.0 ± 4.0
Taurine	1.0 ± 0.5	4.5 ± 1.4

Glutamate was not quantifiable in 3/3 astrocyte conditioned medium tested; Alanine was detected only in 1/3 astrocyte conditioned medium at 32 μM.

Since under specific/pathological circumstances, astrocytes may also release glutamate, (Malarkey and Parpura, 2008), we performed HPLC analysis on astrocytes-conditioned medium, observing an extremely low concentration of glutamate (1.5 ± 0.9 μ M, *n* = 4) in respect to GCM. These data indicate that, in our experimental conditions, glutamate release is a specific feature of glioma cells.

Trying to identify the mechanism of glutamate release from glioma cells, we first put attention to the *X*_*c*_ transport system (Buckingham et al., 2011), analyzing the GCM obtained in the presence of the *X*_*c*_ system blocker sulfasalazine (SAS, 250 μM, 4 h). As shown in Figure 3B, SAS treatment did not reduce the amount of glutamate released in the GCM (94 ± 10% of control; *n* = 4). We further tried to interfere with glutamate release from glioma cells, by blocking glutamate transporters with the competitive, non-transportable excitatory aminoacid transporters (EAATs) blocker β-threo-benzyloxy aspartate (TBOA, Shimamoto et al., 1998). However, GCM prepared in the presence of TBOA (100 μM), showed an increased glutamate level (148 ± 16%, *p* = 0.01; *n* = 4), indicating that in MZC cells EAAT transport is not necessary for glutamate release and rising the suggestion that it may be involved in glutamate uptake (Takano et al., 2001).

Another possible way of glutamate release is linked to osmotic stress (Takano et al., 2005). Since the extracellular solution used for the preparation of GCM, is hypotonic (NES, 300 ± 5 mOsm) compared to glioma culture medium (340 ± 10 mOsm; DMEM), glutamate could be released in response to an osmotic stress. Indeed, it has been reported that several aminoacids are released from neuronal and non-neuronal cells in response to hypotonic stress (Ordaz et al., 2004; Takano et al., 2005; Pasantes-Morales and Vázquez-Juárez, 2012). In particular, glial cells are able to respond to hypoosmotic swelling, with a regulatory volume decrease, through the efflux of chloride, glutamate, and other anions (Kimelberg et al., 1995; Pasantes-Morales and Vázquez-Juárez, 2012). To test this possibility, we analyzed the level of glutamate in the GCM, prepared with a hyperosmotic solution (HyperGCM), having the same osmolarity of the glioma culture medium (~340 mOsm). The concentration of glutamate determined in the HyperGCM by HPLC analysis was 12.5 ± 2.7 μ M (Figure 3C; *n* = 5), similar to that measured in the GCM, suggesting that the osmotic stress is not involved in glutamate release. The same holds for all the identified aminoacids (with the exception of taurine), whose concentrations were similar in the two conditions (Figure 3C).

Altogether, these data indicate that glioma cells release both excitatory and inhibitory neuroactive compounds. Among them, glutamate is released, independently of the most conventional release mechanisms, from glioma cells but not from astrocytes.

### Glycine receptors activation reduces [Cl^−^]_i_ increase by counteracting GCM-induced neuronal depolarization

**Figure 4:**
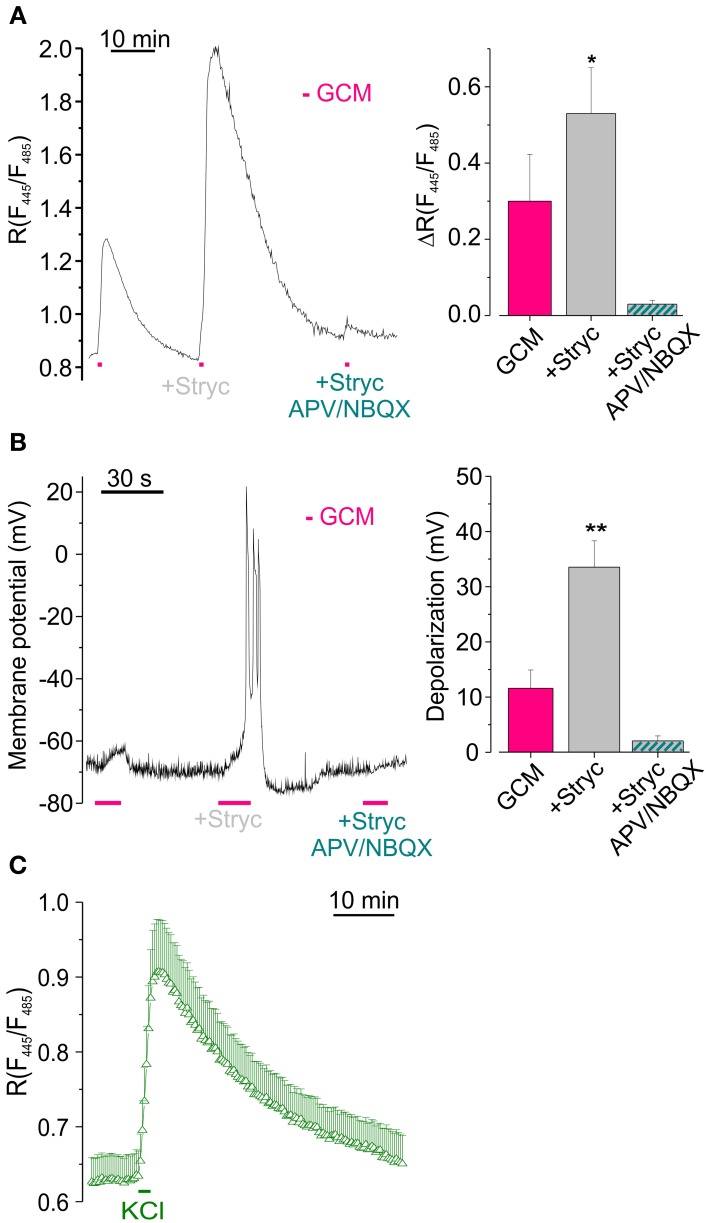
**Glycine receptors activation reduces GCM-induced [Cl^−^]_i_ increase by counteracting neuronal depolarization. (A)** Effect of (1 μM) strychnine on GCM-evoked [Cl^−^]_i_ increase. Left, representative fluorescence trace from a single transfected neuron, monitored during the application of GCM alone, in the presence of strychnine (1 μM) and in the presence of strychnine plus APV (20 μM) and NBQX (10 μM). Right, histogram representing the average *R* increase induced by GCM in GCM alone and in the presence of strychnine (*n* = 9) and in strychnine plus APV/NBQX (*n* = 6). **(B)** Strychnine increases GCM-induced depolarization in hippocampal neurons in current clamp recordings. Left, sample trace from an hippocampal neuron recorded in current clamp during the application of GCM as in **(A)** Note remarkable increase in depolarization in the presence of strychnine. Right, columns represent the depolarization induced by GCM, alone (*n* = 11) or in the presence of strychnine (*n* = 5). **(C)** Time course of *R* increase induced by the application of a modified external medium containing high KCl (20 mM, 1 min; *n* = 18, Δ). ^*^p < 0.05; ^**^p < 0.01.

Since GCM contains a high concentration of glycine, we hypothesized that GCM-induced Cl^−^ influx, may partially occur through Cl^−^ permeable glycine receptors (GlyR). To test this possibility, we applied GCM to hippocampal neurons together with the GlyR antagonist strychnine (1 μM). Contrary to what expected, we observed an enhancement of GCM-induced [Cl^−^]_i_ rise from a control value of Δ*R* = 0.30 ± 0.07 to Δ*R* = 0.53 ± 0.12 (*n* = 9; *p* = 0.05, Figure 4A). Furthermore, GCM-induced responses in strychnine were completely abolished in the presence of APV and NBQX (Δ*R* = 0.03 ± 0.01, *n* = 5). Similar experiments, performed in the presence of gabazine (10 μM), excluded a contribution by GABARs, as no significant changes in Δ*R* were osbserved (0.37 ± 0.05 in control vs. 0.35 ± 0.08 in gabazine, *n* = 7; not shown). These data indicate that when GlyRs are blocked, GCM evokes a stronger increase of neuronal [Cl^−^], entirely dependent on the activation of glutamate receptors.

To investigate whether GCM-induced increase of [Cl^−^]_i_ depended on a change in membrane potential, we exposed cell cultures to GCM during current clamp recordings. GCM application (10–15 s) caused the depolarization of the membrane potential of about 11.4 ± 3.5 mV (*n* = 27, Figure 4B), which was absent in the presence of APV and NBQX (*n* = 4; not shown). Conversely, when applied together with strychnine, GCM-induced a significantly higher depolarization (33.0 ± 4.7 mV, *p* < 0.01; *n* = 20; Figure 3B). Thus, the activation of GlyRs, by glycine released from glioma cells, contrasts GCM-induced neuronal depolarization and this effect is abolished when GlyRs are blocked by strychnine. Consistently, in Cl-Sensor expressing neurons, the reversal potential for chloride was slightly more negative (–73 ± 2 mV) than the resting membrane potential (–66 ± 4 mV), as estimated by gramicidin-based perforated patch clamp recordings (*n* = 6, not shown), indicating that the activation of GlyRs likely induces neuronal hyperpolarization. Again, GCM-induced depolarization in strychnine was abolished by APV and NBQX (2.0 ± 0.9 mV, *n* = 7, Figure 4B), demonstrating that it is entirely dependent on the activation of ionotropic glutamate receptors.

Moreover, when neuronal depolarization was induced by the application of high K^+^ (20 mM, 1 min) an increase in [Cl^−^]_i_ rise was induced with a Δ*R* of 0.29 ± 0.07 (*n* = 18, *p* < 0.01; Figure 4C), supporting the hypothesis that depolarization is the key event for Cl^−^ entrance into neurons.

### Anionic channels and cationic chloride transporters are involved in GCM-induced Cl^−^ fluxes

**Figure 5:**
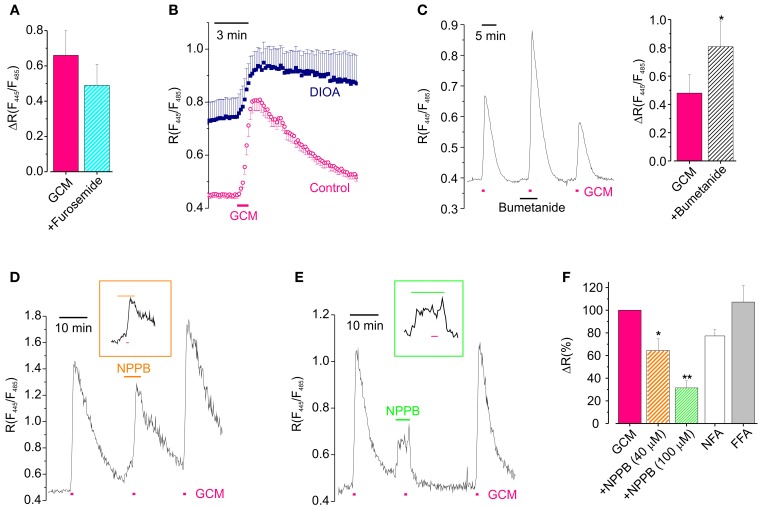
**Cl^−^ channels and transporters are involved in GCM-induced Cl^−^ transients. (A)** Effect of furosemide on the peak amplitude of GCM-induce transients (*n* = 9; *p* = 0.4, paired *t*-test). **(B)** Time course of *R* increase induced by GCM in neurons treated with DIOA (*n* = 9, °) and untreated control neurons (*n* = 7, ■). **(C)** Left, example trace representing the effect of acute application of bumetanide on GCM-induced Cl^−^ transient; right, histogram representing the average effect of bumetanide on GCM-induced ratio increase. **(D)** Example trace of the effect of NPPB (40 μM) on GCM-induced *R* increase; inset, trace with extended time scale representing GCM application in NPPB. **(E)** Effect of NPPB 100 μM; trace and inset as in **(D)**. **(F)** Histogram representing the effect of anionic channels blockers on GCM-induced *R* increase in hippocampal neurons. NPPB 40 μM (*n* = 4) and 100 μM (*n* = 5); NFA (100 μM; *n* = 3); FFA (200 μM; *n* = 5). All data are expressed as the percentage of the control GCM response in the same cells. ^*^p < 0.05; ^**^p < 0.01.

Neuronal depolarization may favor Cl^−^ entry simply augmenting Cl^−^ driving force, or also activating pathways of chloride flux, which are closed at resting potential. Thus we, used a pharmacological approach trying to shed light on the involvement of Cl^−^ channels and transporters in GCM effects. To investigate the role of transporters in GCM-induced Cl^−^ influx, we exposed hippocampal neurons to the cation chloride transporters blocker furosemide (100 μM). In the presence of this compound, the application of GCM-evoked a transient increase in [Cl^−^]_i_ in average not dissimilar in respect to control (*R* = 0.49 ± 0.12 vs. 0.66 ± 0.14; *n* = 9; *p* = 0.4, paired *t*-test; Figure 5A). However, in most of the experiments, the amplitude of *R* increase was reduced or increased by furosemide, suggesting that both KCC2 and NKCC1 could be involved in GCM-induced Cl^−^ flux. In fact furosemide blocks both transporters, which are expressed in cultured hippocampal neurons. We sought to discriminate between the potentially opposite roles of these two Cl^−^ transporters, by using specific antagonists DIOA and bumetanide.

To block KCC2, we incubated Cl-Sensor expressing hippocampal cultures in the presence of DIOA (20 μM; 2 h), performing experiments in the continuous presence of the drug. In these conditions GCM application still induced an increase of *R*, which resulted of Δ*R* = 0.30 ± 0.11 (*n* = 10) compared to slightly higher controls (0.55 ± 0.19, *n* = 8, *p* = 0.25, independent *t*-test). However, as shown in Figure 5B, Cl^−^ did not recover to basal level after GCM washout, indicating that KCC2, although not required for GCM-induced Cl^−^ entry, is necessary for reestablishing Cl^−^ equilibrium. In addition, the basal *R* in cells treated with DIOA was higher than control, indicating that prolonged block of KKC2 leads to accumulation of basal Cl^−^ in neurons (see also Pellegrino et al., 2011). Conversely, when we exposed neurons to GCM in the presence of bumetanide, to block NKCC1, we observed an increase in the amplitude of *R* transients from a control Δ*R* of 0.48 ± 0.13 to 0.81 ± 0.18 (*n* = 8; *p* = 0.03; Figure 5C). This augmented Cl^−^ influx is likely due to the block of NKCC1-mediated rapid Cl^−^ extrusion by reverse transport during intracellular Cl^−^ load (Brumback and Staley, 2008). The block of NKCC1 did not alter the slow recovery after GCM application, indicating that, in our experimental conditions, this transporter is not necessary as KCC2 for the recovery of normal Cl^−^ equilibrium. In addition, the application of bumetanide did not cause significant changes in the basal fluorescence ratio. All together, these data indicate that cation chloride transporters do not play a key role in GCM-induced Cl^−^ entry but their activity may modulate the amount of Cl^−^ entry likely affecting the speed of recovery or changing the driving force of Cl^−^ flux.

It is known that glutamate may lead to neuronal Cl^−^ influx through the activation of different classes of anionic channels (Backus et al., 1998; Van Damme et al., 2003; Slemmer et al., 2004; Inoue and Okada, 2007). Thus, to find out whether anionic channels contributed to GCM-induced neuronal Cl^−^ influx, we tested the effect of the broad spectrum anion channels blocker 5-Nitro-2-(3-phenylpropylamino) benzoic acid (NPPB, 40–100 μM).

NPPB, which is known to block a Cl-driven outwardly rectifying current in neurons (Van Damme et al., 2003), dose dependently reduced GCM-induced increase in [Cl^−^]_i_. At 40 μM it caused a reduction of Δ*R* to 66 ± 10% of control (*n* = 4, *p* < 0.05; Figures 5D,F) and at 100 μM to 31 ± 6% of control (*n* = 9; *p* < 0.01; Figures 5E,F), indicating that NPPB-sensitive anion channels largely contribute to GCM-induced Cl^−^ entry in hippocampal neurons. In addition, in the presence of 100 μM NPPB cells were unable to recover [Cl^−^]_i_ to basal level after GCM application or neuronal Cl^−^ load. This suggests that anionic channels are involved in GCM-induced Cl^−^ entry, as well as, its recovery. NPPB also caused, by itself, an increase in the basal fluorescence ratio (see Figures 5D,E), likely due to Cl^−^ influx, as it was absent when the drug was applied in low external Cl^−^ (8 mM; *n* = 3, not shown).

It has been reported a slight reduction effect of NPPB on AMPA-mediated currents (Van Damme et al., 2003). To ascertain the specificity of its effect the on Cl^−^ flux, we checked whether this drug could affect the glutamate-induced depolarization in current clamp recordings. Glutamate similarly depolarized neurons in control and in the presence of NPPB, respectively, to −10.0 ± 2.2 and to −13.6 ± 6.3 mV (*n* = 5, *P* = 0.5). However, NPPB itself caused a significant depolarization of the neuronal membrane potential of 10.2 ± 2.4 mV (*n* = 8, not shown) which could be the cause of NPPB-induced Cl^−^ influx and of the delayed recovery of basal Cl^−^ level.

To further analyze the involvement of anionic channels, we tested the effect of two other anionic channels blockers niflumic acid (NFA) and flufenamic acid (FFA) on GCM response (Figure 5F). NFA (100 μM) reduced the GCM-induced Cl^−^ transient to 77 ± 6% of control (*n* = 3; *p* = 0.06), while FFA (200 μM) was uneffective (107 ± 14; *n* = 5; *p* = 0.78). NFA, similarly to NPPB, also delayed recovery of [Cl^−^]_i_ after GCM-induced transients (not shown).

Altogether, these data indicate that the application of GCM mainly determines neuronal [Cl^−^]_i_ influx, increasing due to depolarization the driving force for Cl^−^ entry, presumably through anionic channels. Anionic channels and cation chloride transporters are also involved in the recovery of [Cl^−^]_i_ equilibrium after GCM-induced Cl^−^ load.

## Discussion

In the present study, we characterized the effect of neuroactive aminoacids released by glioma cells on neuronal [Cl^−^]_i_, taking advantage of a genetically encoded CFP/YFP-based ratiometric Cl-Sensor (Markova et al., 2008; Bregestovski et al., 2009; Waseem et al., 2010), which was transiently expressed in cultured hippocampal neurons. We observed that acute application of GCM causes a rapid [Cl^−^]_ì_ increase in neurons via glutamatergic mechanism, mimicked by the application of glutamate and largely dependent on NPPB-sensitive anionic channels. Moreover, HPLC and electrophysiological analysis showed that GCM contains a functionally relevant amount of glutamate and other neuroactive aminoacids, including aspartate, glycine, and serine. Glycine, released by glioma cells, counteracts the mechanisms triggered by glutamate, likely contrasting neuronal depolarization.

To date, there is little knowledge available on the mechanisms regulating homeostasis of Cl^−^ in either physiological or pathological conditions, especially for the technical difficulties in the measurement of Cl^−^ fluxes. In this paper, we used Cl-Sensor, a probe with a high sensitivity to Cl^−^ (EC_50_ ~30 mM), that has been demonstrated to be an effective tool for the quantitative estimation of [Cl^−^] in various cellular compartments (Markova et al., 2008; Bregestovski et al., 2009; Waseem et al., 2010). In our study, several lines of evidence support the notion that GCM alters neuronal Cl^−^ equilibrium, causing glutamatergic-mediated Cl^−^ infux in hippocampal neurons: (i) GCM-induced fluorescence increase is abolished in the presence of antagonists of ionotropic glutamatergic receptors; (ii) exogenously applied glutamate evokes similar [Cl^−^]_i_ rise; and (iii) CGM contains glutamatergic agonists at functionally relevant concentration. The ability of glutamate to cause elevation of intracellular [Cl^−^] has been already shown in physiological (Van Damme et al., 2003; Slemmer et al., 2004; Kitamura et al., 2008) and pathological contexts (Inoue and Okada, 2007; Wang and Qin, 2012). Particularly relevant to our study is that excitotoxic stimulation of glutamate receptors may also lead to pH changes (Wang et al., 1994), since Cl-Sensor properties might be influenced by acidification (Markova et al., 2008). However, in our experimental conditions, fluorescence ratio increases reasonably represent [Cl^−^]_i_ accumulation, because the response evoked by application of glutamate disappeared when we strongly decreased Cl^−^ driving force, lowering external [Cl^−^] to 8 mM. Consistently, Waseem et al. (2010) and Pellegrino et al. (2011) illustrated the transient glutamate-induced increase of [Cl^−^]_i_, in spinal neurons and hippocampal neurons transfected with Cl-Sensor.

HPLC analysis on GCM confirmed that MZC cells, exposed to control external medium, release a relevant concentration of glutamate, together with other neuroactive aminoacids, giving results very consistent with those found in microdialysis experiments from glioma and peritumoral space in implanted rats (Behrens et al., 2000). In chromatographic analysis we focused on aminoacids commonly involved in signaling mechanisms, although some of them still need to be ascertained (see Behrens et al., 2000). Apparently, the mechanism of glutamate release from glioma cells is different from the most common ways of glutamate transport or release, being insensitive to *X*_*c*_ system or EAATs inhibition. This is not surprising since *X*_*c*_ system contribution should be limited in the absence of extracellular cystine and EAATs activity is controversial in glioma cell lines (Ye et al., 1999; Buckingham et al., 2011). Our data with TBOA suggest that EAAT transport system is functional in MZC cells, likely participating in glutamate uptake. Although glutamate release from MZC cells is not evoked by osmotic stress, as described in glial cells, we cannot exclude a contribution of anionic channels, possibly activated by other means (Takano et al., 2005). However, the mechanism of glutamate release by MZC cells needs further investigation. Indeed, other typical ways of astrocytic glutamate release, as P2X7 ATP-gated channels, gap junction hemichannels, or vescicular release (Malarkey and Parpura, 2008), could be functional in glioma cell cultures (see Samadani et al., 2007). Notably, the observed glutamate release is a specific feature of glioma cells, being glutamate concentration detected in astrocyte conditioned medium, extremely low in comparison to GCM. It should be considered, alternatively, that the observed difference in glutamate concentration might depend on a more functional astrocytic glutamate uptake rather than on different release properties (Takano et al., 2001).

Indeed, the HPLC results showed that GCM contained relevant concentrations of other aminoacids including glycine, serine, and aspartate and might in various ways interfere with neuronal activity. It is known that aminoacids may be released by glial cells both for osmotic (Ordaz et al., 2004) and signaling functions, several of them being already identified as putative gliotransmitter (Behrens et al., 2000; Cavallero et al., 2009). However, some of these substances might have a specific action during glioma invasion and the circumstances of release from glioma cells deserve further investigation.

Our results show that GlyR activation interferes with the depolarizing effect of GCM. The Cl^−^ influx through GlyRs may act as a shunting mechanism that counteracts the glutamate-induced depolarization of the membrane potential. Depolarization is likely the key event in GCM-induced Cl^−^ influx, as previously proposed for glutamate effects (Backus et al., 1998; Van Damme et al., 2003; Slemmer et al., 2004) and confirmed here by the ability of K^+^ to induce a Cl^−^ rise similar to that observed with glutamate or GCM. It could be discussed that in the presence of strychnine, GCM-induced Cl^−^ accumulation is abolished by GluR antagonists, while a residual Cl^−^ transient is observed when GlyRs are not blocked. These results could indicate a participation of GlyRs to Cl^−^ influx, particularly in cells with depolarized resting potential.

Cation chloride transporters have a minor role in GCM induced Cl^−^ flux, being mainly involved in restoration of Cl^−^ equilibrium after GCM stimulation. The potentiating effect of bumetanide is likely due to the block of NKCC1-mediated rapid Cl^−^ extrusion by reverse transport during intracellular Cl^−^ load (Brumback and Staley, 2008). Alternatively, it is possible to speculate that bumetanide effect on GCM-induced Cl^−^ influx, might reflect an increase in Cl^−^ driving force, caused by bumetanide mediated decrease in [Cl^−^]_i_, (Chabwine et al., 2009). However, this possibility is not supported by our observations.

On the other hand, we show that the depolarization-induced Cl^−^ influx involves NPPB-sensitive anionic channels and a significant increase of the neuronal driving force for Cl^−^. The main concern in the interpretation of our results arises from the lack of specific pharmacological tools for anionic channels. In fact, many of the compounds used to block anionic channels are reported to have unspecific effects and, as shown here, may alter Cl^−^ equilibrium and membrane potential. However, our data are strongly consistent with previous reports, showing that anionic channels, activated by depolarization or swelling, are responsible of neuronal Cl^−^ entry, following glutamate receptor activation (Van Damme et al., 2003; Slemmer et al., 2004; Inoue and Okada, 2007). We speculate that these may include Ca^2+^-, volume- and voltage-activated Cl^−^ channels (Payne et al., 2003; Suzuki et al., 2006; Jentsch, 2008; Deisz et al., 2011). However, other anion permeable channels could be involved, including the high conductance hemichannel Pannexin 1 which is expressed in neurons and is a Cl-permeable and sensitive to NPPB (Ma et al., 2012).

Altogether, our results suggest a mechanism allowing glioma cells to deeply and dynamically interfere with neuronal Cl^−^ homeostasis, through the release of excitatory and inhibitory neuroactive substances. In physiological conditions, a similar inhibitory shunt of glutamatergic responses by Cl^−^ permeable ligand-gated channels, might occur in case of GABA and glutamate corelease, as described in hippocampal mossy fibers (Treviño et al., 2011), or as a consequence of tonic GABA release (Semyanov et al., 2004). The observed shunt is reminiscent of that caused by unconventional GABA release (Koch and Magnusson, 2009), i.e., during intense synaptic activity (Glykys and Mody, 2007). Interestingly, the resulting excitation/inhibition balance can be altered an in epilepsy models, as a results of increased GABAergic inhibition (Treviño et al., 2011). A possible concern in the interpretation of our results is represented by the use of neuronal cultures. In the healthy brain, glutamate is rapidly removed from the extracellular space by astrocytes, preventing extensive or prolonged activation of neuronal glutamate receptors (Danbolt, 2001) and its action may be contrasted by tonic GABA release (Semyanov et al., 2004). Although, it is known that during tumor invasion brain microenvironment is altered (Behrens et al., 2000), the application of GCM might not represent the *natural* concentration profile experienced by neurons. Further *in vivo* studies will be needed to ascertain if the proposed mechanism may support the alterations of Cl^−^ homeostasis, causing the imbalance of inhibitory and excitatory neuronal network properties observed in glioma associated epilepsy.

### Conflict of interest statement

The authors declare that the research was conducted in the absence of any commercial or financial relationships that could be construed as a potential conflict of interest.

## Abbreviations

ACM: Astrocyte conditioned medium
APV: D-2-amino-5-phosphonopentanoic
[Cl^−^]_i_: chloride concentration
CNS: central nervous system
DMEM: Dulbecco's Modified Eagle Medium
DMSO: Dimethyl sulfoxide
EAATs: Excitatory aminoacid transporters
FBS: Fetal Bovine Serum
FFA: flufenamic acid
GABA: γ-aminobutyric acid
GCM: glioma conditioned medium
Glu: glutamate
GluR1-HEK cells: HEK 293 stably expressing the rat flip variant of wild-type glutamate receptor 1
HBSS: Hank's balanced salt solution
HPLC: High Performance Liquid Chromatography
HyperGCM: hyperosmotic glioma conditioned medium
NBQX: 2,3-dihydroxy-6-nitro-7-sulfamoyl-benzo[f]quinoxaline-2,3-dione
NES: normal external solution
NFA: niflumic acid
NPPB: 5-Nitro-2-(3-phenylpropylamino)benzoic acid
OPA: *o*-phthaldialdehyde
SAS: Sulfasalazine
TBOA: DL-*threo*-β-Benzyloxyaspartic acid
TTX: Tetrodotoxin.
